# Curcumin for the Management of Periodontitis and Early ACPA-Positive Rheumatoid Arthritis: Killing Two Birds with One Stone

**DOI:** 10.3390/nu10070908

**Published:** 2018-07-16

**Authors:** Eleni Asteriou, Athanasios Gkoutzourelas, Athanasios Mavropoulos, Christina Katsiari, Lazaros I. Sakkas, Dimitrios P. Bogdanos

**Affiliations:** Department of Rheumatology and Clinical Immunology, Faculty of Medicine, School of Health Sciences, University of Thessaly and University General Hospital of Larissa, 41110 Larissa, Greece; eleniaster91@gmail.com (E.A.); gkoutzou@med.uth.gr (A.G.); mavropoulos_thanos@hotmail.com (A.M.); cgk2005@gmail.com (C.K.); lsakkas@med.uth.gr (L.I.S.)

**Keywords:** autoantibody, gingivitis, infection, periodontitis, rheumatoid arthritis, rheumatic diseases

## Abstract

We propose curcumin as a preventive measure to avoid/manage periodontitis (PD), and as a natural immunosuppressant for rheumatoid arthritis (RA). PD, mainly caused by *Porphyromonas gingivalis* forming biofilm and leading to tooth decay, is a major public health issue and a risk factor for the development of RA in humans. *P. gingivalis* is able to trigger experimental autoimmune arthritis in animal models and in humans can induce citrullinated peptides, which not only are a source of anti-citrullinated antibodies (ACPAs), but also participate in autoreactive responses and disease development. Curcumin appears to have efficient anti-bacterial activity against *P. gingivalis* infection and biofilm formation. In addition to antibacterial, anti-oxidant, and anti-inflammatory action, curcumin exerts unique immunosuppressant properties via the inhibition of Th17 pro-inflammatory responses and promotion of regulatory T cells, thus suppressing autoimmunity. We introduce curcumin as a natural product for the management of both PD and RA-related autoreactivity, possibly also as a preventive measure in early RA or individuals at high risk to develop RA.

## 1. Introduction

Rheumatoid arthritis (RA) is an autoimmune rheumatic disease characterized by immune-mediated joint inflammation, which leads to joint destruction, loss of joint function, and disability, if left untreated [1,2,3]. In fact, joint erosion can develop early during the first three months of the disease in 25% of RA patients and mostly during the first two years of the disease [4].

The immunopathogenesis of RA has been extensively investigated and it is well established that genetic factors, predominantly HLA-DRB1 shared epitope (HLA-DRB1SE) alleles [5,6], epigenetic, and environmental factors are involved in the development of the disease [1,2,7,8,9,10]. In immunopathological terms, proinflammatory Th1 cells and B cells and proinflammatory soluble mediators (TNFα, IFNγ, IL-6) are involved in the pathogenesis of the disease [11]. A better understanding of the underlying mechanisms of the disease has led to its therapeutic management with synthetic disease-modifying antirheumatic drugs (sDMARD) and novel biological agents that target specific molecules involved in disease pathogenesis, which can prevent joint damage and disease progression, and improve disease prognosis [2,9,11,12,13]. Since these therapies can have potentially toxic side effects, it is very important for practicing physicians to diagnose the disease early and accurately, especially more aggressive forms, in order to select the appropriate treatment [14,15].

## 2. Curcumin as a Remedy for the Treatment of Rheumatoid Arthritis: Data from Animal Studies

**Figure 1:**
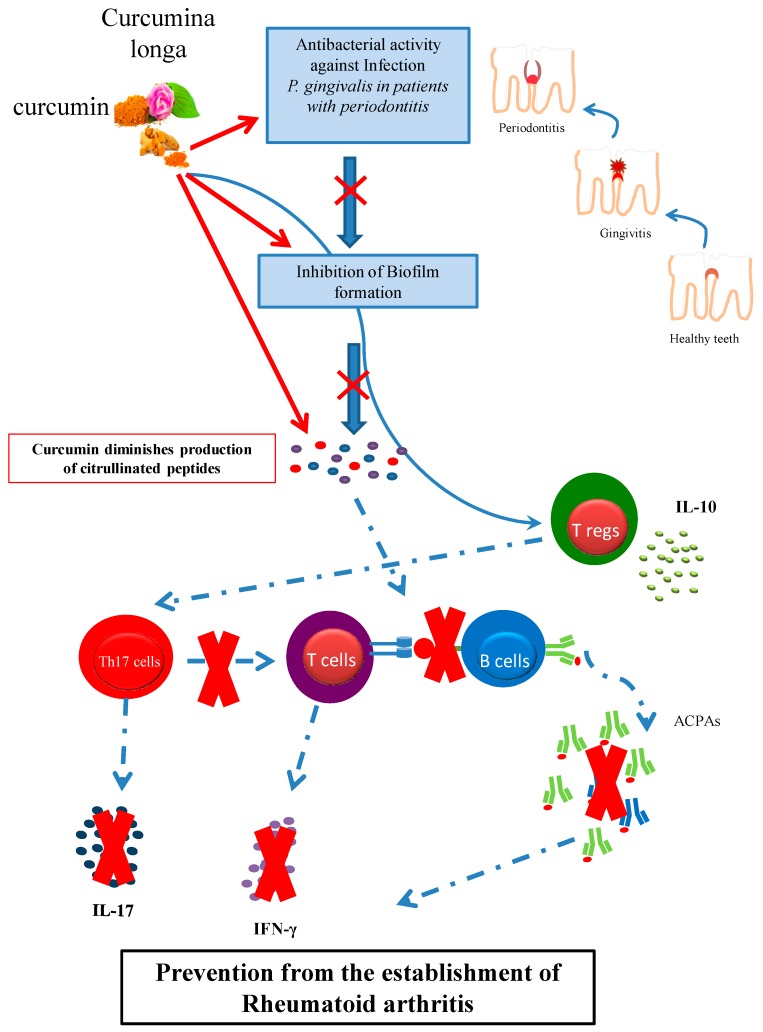
Curcumin may play a significant role in preventing from rheumatoid arthritis through its antibacterial action against *P. gingivalis* infection and biofilm formation in patients with periodontitis; and modulation of the proinflammatory immune response, such as inhibition of Th17 cells and enhancement of IL-10 producing regulatory T cells.

Curcumin, a traditional remedy and major curcuminoid found in the spice turmeric, has been used for centuries for treating chronic inflammatory diseases [16,17,18,19]. Recent studies have demonstrated the decisive role of curcumin in treating autoimmune diseases [19,20,21,22], including experimental autoimmune arthritis, the animal model of rheumatoid arthritis (RA) [23,24,25,26,27,28,29,30,31,32,33,34,35,36,37,38]. How curcumin exerts its beneficial effect in experimental arthritis is under investigation [23,24,25,26,27,28,29,30,31,32,33,34,35,36,37,38]. It appears that curcumin has the ability to decrease pro-inflammatory Th1 and Th17 cells and increase regulatory T cells [23,24,25,26,27,28,29,30,31,32,33,34,35,36,37,38,39] (Figure 1). Recent evidence shows that reciprocal regulation of Th17/Treg cells (decreasing Th17 cells and increasing Tregs) by administration of IL-10-producing cells (Bregs), can suppress collagen-induced arthritis (CIA) and thus IL-10-producing Bregs can be considered an attractive therapeutic strategy for T cell-mediated autoimmune rheumatic diseases (ARDs) such as RA [40,41,42]. Currently, there is no available approved medication able to efficiently modulate the Th17/Treg/Breg balance in vivo in RA. Curcumin, as a bioactive immunomodulatory agent, has well-documented actions against proliferating lymphocytic populations [43], yet its role in modulating Th17/regulatory cell responses in ARDs remains largely unexplored. However, there are studies on murine models of experimental arthritis investigating the role of curcumin; these are summarized in Table 1.

### 2.1. Experimental Arthritis

The CIA model is one of the best-characterized murine models for RA. In genetically susceptible murine strains, such as C57BL/6 mice, the administration of type II collagen (CII) causes a robust and sustained T-cell response to administered CII, leading to synovitis and erosion that histologically resemble RA [44]. It is also a significant model for studying Th17 responses [45] as symptoms of CIA are markedly suppressed in mice lacking IL-17 (IL-17−/−mice) [45,46]. Paradoxically, the effect of curcumin on IL-17 production in CIA induced in C57BL/6 mice has not been thoroughly investigated. Cong et al. [47] using C57BL/6 (B6) and B6.RAG-2−/−mice have demonstrated that curcumin could induce tolerogenic bone marrow-derived dendritic cells that promote the differentiation of intestinal Tregs [47]. Some evidence of Th17 cells was documented in C57BL/6 mice with a mutation in the Foxp3 gene scurfin (scurfy mice) that develop polyendocrinopathy and enteropathy X-linked (IPEX) syndrome, a lethal autoimmune disease [48]. Curcumin ameliorated IPEX syndrome by inhibiting Th1/Th2/Th17 responses [41]. Scurfy mice on a curcumin diet survived four times longer (92.5 days) compared to scurfy mice fed a normal diet (23 days), and in in vitro experiments curcumin decreased the production of cytokines IFN-γ (Th1 cytokine), IL-4 (Th2 cytokine), and IL-17A (Th17 cytokine) in CD4+ T cells [41]. 

Okamoto et al. [35] has reported that curcumin treatment inhibited IL-17 production in vitro and ameliorated CIA in DBA/1 J mice. A significant anti-arthritic effect of curcumin has also been previously demonstrated in CIA induced in DBA/1 J mice but no information has been given on the effects on Th17 cells. Huang et al. [37] injected mice intra-peritoneally with curcumin and found decreased levels of B cell-activating factor (BAFF), IFN-γ, and IL-6 in serum and their production by spleen cells but gave no information on IL-17 production [37]. DBA/1 mice immunized with CII and treated with curcumin every other day for two weeks also demonstrated reduced clinical arthritis scores, associated with reduced expression of TNF-alpha and IL-1beta in the ankle joint, and decreased levels of IgG2a in serum [36]. Orally administrated curcumin also suppressed the production of matrix metalloproteinase (MMP)-1 and MMP-3 and ameliorated CIA [38]. Curcumin can also attenuate collagen-induced inflammatory responses through the “gut–brain axis” by modulating the function of the cholinergic system [23]. 

Funk et al. [34] were the first to report the anti-arthritic effect of complex turmeric extracts containing curcuminoids in experimental arthritis induced in Lewis rats [34]. The anti-inflammatory effect of the tetramethylpyrazine, resveratrol, and curcumin (TRC) combination in acute and chronic inflammation was reported in vivo in CIA induced in rats [28]. The TRC combination could inhibit the production of TNF-α, IL-1β, and IL-6 in the serum but, again, no information was available for IL-17 levels [28]. In addition, curcumin administered via the intravenous (IV) or oral route had a therapeutic effect similar to methotrexate on adjuvant-induced arthritis in rats, an effect associated with decreased levels of TNF-α and interleukin-1β in both synovial fluid and serum [25].

### 2.2. Experimental Periodontitis

In experimental periodontitis (PD), an effect of curcumin on systemic Th17 responses, gingival IL-17A expression, Retinoic Acid Receptor-Related Orphan Receptor γt, and alveolar bone loss has recently been described [49].

### 2.3. Experimental Autoimmune Encephalitis

More data on curcumin and Th17 responses are available from studies in experimental autoimmune encephalomyelitis (EAE), a model for multiple sclerosis (MS). In EAE, strong evidence suggests that IL-17-producing T cells play a dominant pathogenic role. In EAE induced by the MOG-peptide 35–55 in C57BL/6 mice, CD4+ Th17 cells are present both in the periphery and in the inflamed central nervous system [50] and amelioration of EAE by curcumin treatment was through inhibition of IL-17 production [39]. Further studies indicated that dietary curcumin inhibited the differentiation of pro-inflammatory Th1/Th17 cells in vivo during encephalomyelitis and instead promoted Th2 cells [51]. Curcumin appeared to silence IL-23/Th17-mediated pathology in EAE by enhancing HO-1/STAT3 interaction in dendritic cells (DCs) [51].

## 3. Human Rheumatoid Arthritis: Data from Clinical Trials

Clinical data in human RA have started to emerge. Though limited, the findings from two small clinical trials are encouraging [52,53]. The first small randomized clinical trial [52] enrolled 45 patients with active RA into three groups, receiving curcumin (500  mg) alone, diclofenac sodium (50  mg) alone, or curcumin and diclofenac in combination. The primary endpoint was good or moderate Disease Activity Score (DAS) 28 response, and the secondary end point was C-reactive protein (CRP) levels at week 8 post-treatment. Curcumin administration was safe and no adverse events were reported. Patients in all three treatment groups showed statistically significant changes in their DAS28 scores. DAS28 was reduced from 6.40 at baseline to 3.55 in the curcumin-alone group, and from 6.72 to 3.89 in the diclofenac-alone group. However, CRP levels were reduced by 52% in the curcumin-alone group but were not reduced in the diclofenac-alone group [52]. Another small randomized, double-blind, placebo-controlled study [53] evaluated the efficacy of curcumin compared with that of a placebo in RA patients with medium disease activity and rather low CRP levels. A novel curcumin matrix formulation, with 10-fold bioavailability compared to unformulated 95% curcumin, was given to all participants. Twelve patients in each group received the placebo, 250 or 500 mg of the curcumin product twice daily for 90 days. Curcumin was well tolerated without adverse effects. At the end of the study, there was a significant DAS28 improvement and ACR20 response in a high percentage of patients who received curcumin at either the 250 or 500 mg dose. In the curcumin groups, the disease improvement was accompanied by a significant decrease in markers of inflammation (CRP and erythrocyte sedimentation rate, ESR), and rheumatoid factor (RF) levels [53]. These results are encouraging and should be confirmed in larger trials, which will also address the safety profile of long-term usage of curcumin or a supplement’s tolerance. In addition, the reduction of RF levels to be found in such a short time post-treatment is unexpected and requires confirmation. There are no reports on the effect of curcumin on ACPA levels. Nevertheless, these findings support the notion that curcumin may indeed have beneficial effects not only in experimental models of arthritis but also in patients with RA. The work performed in animals has hinted at the pathophysiological pathways that could account for curcumin’s advantageous effects. Another point to be considered is the long-term safety profile of curcumin at pharmacological dosing, which is not known at present.

## 4. Curcumin’s Antibacterial, Antiviral, and Antifungal Action: A Mode to Prevent Pathogen-Induced RA?

A number of studies have investigated the antibacterial, antiviral, and antifungal activity of curcumin [54]. Most in vitro studies clearly demonstrate extensive antimicrobial activity of curcumin [54]. Many in vitro and in vivo studies have provided data in support of curcumin’s inhibitory effects [55]. A characteristic example of promising data stems from studies investigating the inhibitory effect of curcumin in *Helicobacter pylori*, whereby curcumin alone or more efficiently in combination with conventional antibiotics diminishes the symptoms of gastritis and eradicates the bacterium [56,57]. Similar results were obtained in viral pathogens including herpes viruses, placing curcumin on the long list of natural antiviral compounds [58,59,60]. Candida species, *Paracoccidioides brasiliensis*, and other fungi were also inhibited by curcumin [61].

Despite these promising data, more clinical trials are needed to establish the potential clinical use of curcumin [62]. Currently, curcumin-based compounds are in use not only as part of traditional medicine, but also as dietary supplements in several countries, including China, India, Japan, Korea, South Africa, Thailand, Turkey, and the United States, and the list of countries increases at a fast pace.

To better understand why the antimicrobial activity of curcumin may be beneficial for a typical autoimmune disease like RA (Figure 1), we must first understand the role of specific microbes in the breaking of tolerance of this disease [8,63].

## 5. Rheumatoid Arthritis, Anti-Citrullinated Peptide Antibodies, Citrullination, and Periodontitis

The serological marker of RA is the presence of high-titer autoantibodies (autoabs), namely rheumatoid factor (RF) and abs against citrullinated peptides (anti-CCP abs, ACPAs) [1,64,65,66]. ACPAs appear years before the clinical onset of RA. Of clinical importance, the presence of peptide-specific ACPAs predict the future development of the disease in patients with undifferentiated arthritis [4,65,67,68], raising the question of whether such autoantibodies or ACPA-producing plasmocytes play a pathogenic role in the development of RA [69,70,71]. Along these lines, several studies have clearly demonstrated that ACPA is the most reliable prognosticator of radiographic progression in RA [72].

Prompt therapeutic intervention at very early disease stages, a period known as the “window of opportunity,” can slow disease onset and progression [14,15]. Of relevance, there is an ongoing debate among investigators as to how we can manage and whether we can/must intervene in anti-CCP (ACPA) seropositive patients with no recognizable clinical arthritis [73]. 

Nevertheless, upon the discovery of ACPAs, a better understanding of the immunopathogenesis of RA has been gained [65,74]. ACPAs recognize few citrullinated peptides in early preclinical disease states, but over time the number of recognized citrullinated peptides increases; such an increase is accompanied by elevated levels of pro-inflammatory cytokines and finally clinical disease [70,75]. The fact that the presence of ACPAs, particularly those against specific citrullinated peptides, is associated with more aggressive/severe disease supports the notion that such peptides are indeed makers of the disease rather than markers of it [11,65,73,76].

Citrullination is a post-translational modification of proteins caused by peptidyl arginine deiminases (PADs) [77,78]. At the experimental level, it has been shown that citrullination can form neoantigens that activate T cells. Such T helper (Th) cells can offer antigen-specific help to B cells to differentiate into ACPA-producing plasma cells [8,79,80]. Convincing data have shown that citrullination amplifies the affinity of peptides to HLA-DRB1SE alleles [81,82]. Of interest, T cells can also recognize PAD. Such an ability of T cells has a clear implication, namely the capacity of T cells to help plasmocytes produce Abs against hapten/carrier citrullinated proteins bound to PAD [83].

The extent to which pathogens play a role in the development of RA started to unfold when it became clear that citrullination induced by *Porphyromonas* (*P. gingivalis*), a pathogen of PD, can be a source of citrullinated peptides (such as neoantigens) that can then break immunological tolerance [63,84,85,86,87]. *P. gingivalis* produces gingipains, proteases that cleave proteins at peptidyl arginine, and PAD (PPAD) that preferentially citrullinates C-terminal arginine, thus creating neoantigens [88]. There is evidence of *P. gingivalis* infection years before the onset of clinical RA [87,89].

There is an association between *P. gingivalis* infection and ACPA-positive RA [8,63,90,91]. Furthermore, in ACPA-positive RA an interaction between markers of *P. gingivalis* infection, smoking, and HLA-DRB1SE was reported [92]. ACPA to citrullinated α-enolase peptide 1 (CEP1), an immunodominant peptide in RA, showed high homology with α-enolase from *P. gingivalis* and cross-reacted with citrullinated recombinant *P. gingivalis* enolase [93]. *P. gingivalis* inoculation of mice caused PPAD-dependent exacerbation of CIA [94]. In addition, oral inoculation of *P. gingivalis* in HLA-DR1 transgenic mice transiently increased Th17 cells in regional lymph nodes and peripheral blood, induced a massive increase in proinflammatory cytokines, and exacerbated CIA [95]. *P. gingivalis* can also affect inflammation through gut microbiota (discussed later) [96,97].

Another causative agent of PD, *Aggregatibacter actinomycetemcomitans (A. actinomycetemcomitans*), also causes citrullination of human proteins through the production of leukotoxin A [84,98]. Furthermore, HLA-DRB1SE is associated with ACPA only in RA patients exposed to *A.actinomycetemcomitans* [98]. Epstein–Barr virus (EBV), which infects epithelial cells and B cells, also causes ACPA production. ACPAs against peptides derived for EBV nuclear antigen (EBNA)1 (PCV1) and EBNA2 (PCV2) cross-react with human citrullinated proteins [99]. In addition, ACPAs against PCV1 and PCV2 and ACPAs against histone-4-derived citrullinated peptide (HCP1) and HCP2 appear years before the onset of clinical RA and predict with a high risk ratio (OR = 8 to 19) the subsequent development of RA [90]. EBV is also a cause of PD [100].

Newly diagnosed, untreated RA patients show a high prevalence of PD and significant changes to their oral microbiome, compared to those without RA [101]. Also, microbiome changes seen in saliva and dental samples appear to be restored, at least in part, after efficient treatment of the underlying disease [102].

*P. gingivalis* can cause arthritis through changes of gut microbiota. Orally administered *P. gingivalis* changed the gut microbiome with a decrease in *Bacteriodetes* phylum, increased Th17 cells in mesenteric lymphocytes, increased intestinal permeability, and aggravated CIA [96,97,103]. *P. gingivalis* DNA was also detected in synovial tissue from RA patients. More interestingly, *P. gingivalis* DNA in synovial tissue was detected more frequently in HLA-DRB1*04-positive than HLA-DRB1*04-negative RA patients [104]. These findings suggest that molecular mimicry between dysbiotic bacteria and humans may operate in RA as well. The proiflammatory milieu of periodontitis exacerbates arthritis as DNA from periodontopathogenic bacteria stimulates macrophage IL-6 and TNFa production [105].

PD and smoking, the two known environmental risk factors for RA, argue in support of the concept that an immune response mounted at mucosal sites provides the impetus for the initiation of a series of events that culminate in losing immune tolerance and the perpetuation of autoreactive responses leading to RA [106,107,108]. An excellent Swedish study in twins (from the Swedish Twin Registry) has shown that among 12,590 twins, 350 (2.8%) had ACPA (including 124 (1% of the total or 35.2% of the ACPA positive)) [109]. A clear association was found between smoking and HLADRB1SE and the ACPAs’ presence [109]. The authors concluded that environmental and lifestyle (e.g., smoking, PD) rather than genetic factors may play a more important role in the production of ACPAs [109].

Gingival grevicular fluid collected from the space between the tooth and gingival mucosa from patients with PD, not only shows marked citrullination, but also mirrors the hypercitrullination in RA joints; on the other hand, it shows minimal citrullination in healthy individuals without PD [98].

PD is highly prevalent among adults in most developed countries around the globe [110]. It is a chronic disease associated with the development of a pathogenic bacterial biofilm of dental plaque. This bacterial biofilm is one of the most diverse microbial ecosystems within the human body, involving approximately 700 different bacterial species, the most prominent of which are *Porphyromonas gingivalis*, *Prevotella intermedia*, and *A. actinomycetemcomitans* [111]. The encounter of the host’s immune system with these pathogenic bacteria is responsible for the initiation of an immune-mediated inflammation, which ultimately leads to the destruction of periodontal tissues and the tooth. PD is a major public health issue affecting approximately 15% of the adult population worldwide. The socioeconomic impact of the disease is huge and attempts to efficiently manage the disease are still lacking [110]. Surgical intervention, scaling, and root planing accompanied by antibiotic therapy are widely used measures to handle PD. However, their effect is limited, short-term, and may have adverse side effects, the most profound being antibiotic resistance in those undergoing long-term antibiotic treatment [112]. Thus, there is an agreement among investigators that the most efficient means must be non-invasive, preventive measures to avoid the establishment of PD and its deleterious effects via control of the infection and the development and biofilm formation. Towards this, several dietary compounds/supplements have been suggested as efficient and safe, such as plant-originated polyphenol extracts [113], which exert antibacterial (and anti-oxidant or anti-inflammatory), in particular anti-*P. gingivalis* activity, such as cranberries, gallic acid, quercetin, naringin, and, more recently, curcumin.

## 6. Prevention of Early RA: Fighting Periodontitis

Since directly or indirectly citrullinated peptides, ACPAs, and autoreactive lymphocytes specific for such peptides are involved in the pathogenesis of RA, preventive measures must be taken to control or, even better, prevent the development of the vicious circle that places citrullinated peptides as the key player [14,15,64,65,73,114].

If PD induced by *P. gingivalis* or other pathogens is a key factor participating in the loss of tolerance that characterizes early RA [85,89,91,94,104,107,115,116], a key preventive measure would be efficient treatment of PD, i.e., infection by *P. gingivalis* or other PD-related oral pathogens (Figure 1). To this end, lessons must be learned from the work performed on this topic by researchers in the past [117,118,119,120,121,122,123,124,125,126,127,128,129].

### Curcumin and Oral Hygiene: Curcumin Inhibits the Growth of P. gingivalis and Prevents Periodontitis

Following the above argument, an obvious intervention to reduce the risk of ACPA production is to maintain good oral hygiene and treat PD. The rationality of this approach is to stop feeding autoantigens. It becomes apparent that remedies, such as curcumin, could be used as part of preventive measures, especially if they have an effect on the management of PD. Indeed, investigators have studied the effect of curcumin in PD, mainly its ability to inhibit *P. gingivalis* [130]. The lipopolysaccharide (LPS) of *P. gingivalis* stimulates cytokine secretion in immune cells, and has been considered a major cause of inflammation in PD. Macrophages are prominent cell subsets at periodontal sites of inflammation and the effect of curcumin on macrophages stimulated with *P. gingivalis* LPS has been studied [117]. TNF-alpha and IL-1beta expression was inhibited in a dose-dependent manner when the murine macrophage RAW264.7 cell line was pre-treated with various concentrations of curcumin and stimulated by *P. gingivalis* LPS [117]. Also, curcumin can inhibit *P. gingivalis* LPS-induced COX-2 expression, mainly due to the inhibition of the NF-κB pathway [119]. Curcumin also downregulated NF-κB and NF-κB-regulated genes (vascular endothelial growth factor, matrix metalloproteinase 9, cyclo-oxygenase-2) in an orthotopic mouse model of pancreatic cancer [131].

Curcumin can exert antibacterial activity on a series of periodontopathic bacteria, including *P. gingivalis*, *Prevotella intermedia*, *Fusobacterium nucleatum*, and *Treponema denticola.* Bacterial growth was inhibited even at very low concentrations of curcumin, as 20 μg/mL curcumin inhibited the growth of *P. gingivalis* biofilm formations by >80%, suggesting that, at least in vitro, this nutrient can be a potent agent for preventing periodontal diseases [130]. Amongst polyphenols, both curcumin and quercetin are able to alter the architecture of mature multi-species biofilms; however, only curcumin-treated biofilms display significantly reduced metabolic activity [132]. Local curcumin was clinically effective and reduced the microbial load in patients with chronic PD [124].

## 7. Unresolved Issues

Curcumin, the most bioactive curcuminoid of turmeric, has relatively poor oral bioavailability in humans due to low solubility in aqueous solvents, poor stability and absorption, and rapid elimination from the systemic circulation [133]. Such disadvantages may limit its therapeutic application, including fighting or preventing the development of RA. Multiple heterogeneous approaches have been applied to overcome such obstacles, involving the development of curcumin-based nanoparticle formulas, curcumin structure modifications, metabolism inhibitors-based administration, and the development of curcumin prodrugs to improve bioactivity and bioavailability and to optimize its antimicrobial, anti-oxidant, and anti-inflammatory potential [134,135]. This may assist efforts to fight PD, as well as prevent RA, especially in individuals at high risk, such as those who carry susceptible genes (i.e., the HLA–DRB1SE genes). The data are still limited and safe conclusions are difficult to draw. An increasing number of reports, mainly on the Internet, suggest that turmeric (alone or in combination with coconut oil) as a surplus for efficient whitening of teeth, appears attractive, but the data are still lacking. Nevertheless, natural compounds such as turmeric’s curcumin, which convincingly show both RA-related anti-bacterial activity and immunosuppressant/regulatory action, are ideal candidates to focus on in the years to come.

## Figures and Tables

**Table 1:** Curcumin’s biological effects in animal models of experimental arthritis.

Animal Model	Biologic Effect I	Biologic Effect II	Refs
CIA-rat model	Suppressed the inflammatory response and attenuated CIA by targeting the “gut–brain axis”	Increased vagus nerve function directly correlated with the activity of the cholinergic anti-inflammatory pathway	[23]
CIA-rat model	Anti-arthritic efficacy through somatostatin generation via cAMP/PKA and Ca (2+)/CaMKII signaling pathways in the small intestine	Oral administration induced dramatic amelioration of arthritis symptoms When injected intraperitoneally, anti-arthritic effects were lost	[24]
CIA-rat model	Therapeutic effect on RA similar to methotrexate when injected intravenously	Curcumin formulated into oil–water nanoemulsions (Ns) overcame the low oral bioavailability and maintained anti-arthritic potential	[25]
CIA-rat model	Potentiated the anti-arthritic effect of prednisolone	Pronounced beneficial effect on joint swelling, leucocyte count, and biochemical parameters compared with prednisolone	[26]
CIA-rat model	Synergistic activity with methotrexate in ameliorating induced arthritis	Reduced hepatotoxicity in experimental animals	[27]
CIA-rat model	Anti-inflammatory effect in vivo combined with tetramethylpyrazine, resveratrol	Combination significantly reduced paw swelling in acute paw swelling and alleviates the damage in ankle joints, cartilages, and fibrous tissue	[28]
CIA-rat model	Milk-based formulation of curcumin prevented inflammation	increased the bioavailability of curcumin for achieving maximum effectiveness	[29]
CIA-rat model	Topical application of curcumin in combination with emu oil ameliorated induced arthritis	Curcumin–emu oil combination significantly reduced levels of pro-inflammatory mediators	[30]
CIA-rat model	Suppressed pannus formation process that occurred in the articular cartilage of the CIA joints	Insignificant differences of curcumin group compared to betamethasone treated group	[31]
CFA-Induced monoarthritis rat model	Attenuated pain hypersensitivity	Ameliorated spinal neuroinflammation, decreased production of inflammatory mediators in primary cultured astrocytes and microglia	[32]
CFA-Induced monoarthritis rat model	Loaded in solid lipid nanoparticles ameliorated adjuvant-induced arthritis	Attenuated inflammatory and immunomodulatory cascades	[33]
(SCW)-induced arthritis rat model	Prevented joint inflammation	In vivo anti-arthritic efficacy of an essential oil-depleted turmeric fraction	[34]
CIA-DBA/1 mouse model	Inhibited IL-17 production	Decreased the clinical symptoms of CIA	[35]
CIA-DBA/1 mouse model	Suppressed inflammatory response by inhibiting pro-inflammatory mediators	Downregulated clinical arthritis score, and the proliferation of splenic T cells	[36]
CIA-DBA/1 mouse model	Protected against collagen-induced arthritis via suppression of BAFF production	Decreased serum levels of IFN-γ and IL-6, suppressed STAT-1 phosphorylation and nuclear translocation	[37]
CIA- DBA/1 mouse model	Suppressed production of matrix metalloproteinases	Inhibited activation of the PKCdelta/JNK/c-Jun pathway in synoviocytes and chondrocytes	[38]
